# Physicochemical Properties of Explanted Silicone Oil After Use as an Intraocular Tamponade

**DOI:** 10.1167/tvst.11.2.3

**Published:** 2022-02-02

**Authors:** Maximilian Hammer, Sonja Schickhardt, Donald J. Munro, Alexander Scheuerle, Christian S. Mayer, Gerd U. Auffarth

**Affiliations:** 1 David J Apple Laboratory for Vision Research, Heidelberg, Germany; 2 University Eye Hospital Heidelberg, Heidelberg, Germany

**Keywords:** amotio retinae, emulsification, emulsion, emulsion stability, silicone oil, surface tension, zeta potential, viscosity

## Abstract

**Purpose:**

We studied the effects of exposure to biological media within the eye, such as contamination with lipophilic and amphiphilic substances, on the physicochemical parameters of silicone oil used as an intraocular tamponade.

**Methods:**

We removed silicone oil with visible emulsification from 15 patients and measured each sample for shear viscosity and surface tension. We induced in vitro emulsification with balanced salt solution. Using the zeta-potential, we evaluated the emulsion droplet's electrochemical stability. We repeated all experiments in a control group of unused oil. Electrochemical stability and viscosity were additionally measured in oils with high-molecular-weight components.

**Results:**

We recovered silicone oils implanted between 30 and 506 days (mean, 196 days). Viscosity did not differ between explanted and control groups. Surface tension and zeta potential remained unchanged (*P* = 0.61 and *P* = 0.84, respectively). All oils showed a significant correlation of viscosity with temperature (*P* < 0.01 for all). Oils with added high-molecular-weight components showed a lower emulsion stability.

**Conclusions:**

Prolonged contact to hydrophilic biological media does not alter high-viscosity silicone oil's physicochemical parameters. During typical durations of intraocular use, lipophilic and amphiphilic molecules had no deleterious effect. The addition of high-molecular-weight components might decrease the silicone oil's electrochemical emulsion stability, possibly easing the confluence of emulsion droplets.

**Translational Relevance:**

Although the physicochemical parameters of silicone oils are not altered after clinically relevant durations within the eye, emulsion stability significantly differs between oil types.

## Introduction

Silicone oil is a liquid containing polymerized siloxane with organic side chains. It has a high thermal stability, is hydrophobic, and supposedly chemically inert. The use of silicone oils as intraocular tamponades dates from Armaly and separately from Cibis et al, both back in 1962.^1^^,^^2^ Today, implanting silicone oil as a long-term tamponade is widespread and accepted. However, the oil can occasionally and unpredictably emulsify at the interface with its hydrophilic intraocular environment. The emulsification poses a challenge for the clinician. Eyes treated with silicone oil that emulsifies can show up to 72% long-term complications, including corneal decompensation, band keratopathy, chronic elevations of intraocular pressure, opacification of the crystalline lens, retinopathies, and optic neuropathy.^3^^–^^8^ The time course is variable, but generally, emulsification appears about 5 months after surgery. If the oil is not removed, the process can progress to complete emulsification where not only the interface has emulsified, but all of the oil is emulsified, and an opaque formation of oil-in-water emulsion droplets fills the vitreous cavity.^9^^,^^10^

The emulsification is related to a breakdown of the unified silicone oil bubble and breakdown of the bubble's cohesion, as well as changes in the mechanics of the interaction with the surrounding tissues^11^^–^^13^; and the dissolution in the oil of lipophilic and amphiphilic molecules derived from the surrounding blood and ocular tissue. This factor is particularly thought to increase the tendency to emulsification.^4^^,^^14^

That previous work was conducted mainly in vitro. We decided to take a different approach. We investigated how silicone oil is modified by prolonged implantation where it is in contact with the eye's microenvironment including possible contamination of the oil with host lipophilic and amphiphilic metabolites,^4^^,^^14^^–^^17^ that are naturally present in the vitreous cavity.

Our aim was to examine the postimplantation physicochemical properties of silicone oil. We hypothesized that, compared with new oil, explanted oils would (in line with current literature on in vitro research) show decreased surface tension because, during the time in the eye, the oil had been in proximity to biological tissue. At the same time, we predicted that emulsion droplet stability, measured by zeta potential, would increase.

## Materials

### The Collection of Silicone Oil

We recovered silicone oil from the eyes of 15 patients with visible emulsification. All patients included in our study had surgery with documented use of one type and brand of silicone oil, Siluron 5000 (Geuder AG, Heidelberg, Germany). All explantations were performed by one experienced surgeon (C.M.), removing the oil from the eye together with the balanced salt solution (BSS) that was used as a washout within a syringe. After surgery, the syringe and its contents was stored in an upright position. Within 15 minutes, the two media separated, silicone oil floated on top of the BSS, and a clear interface was visible between the two. We carefully harvested the oil from the syringe and discarded the BSS, by repeated use of a 1-mL pipette: 15 samples of roughly 6 mL of oil per sample were collected in this way. Three samples of new and unused Siluron 5000 in a 10-mL syringe were used as a control group throughout all experiments.

Shear viscosity and zeta potential were additionally assessed for four other oil types, new and unused oils with high-molecular-weight components (HMWCs), namely Siluron 2000, Siluron Xtra, Densiron 68 and Densiron Xtra (all from Geuder AG).

Table presents the different compositions and characteristics of the five types of oil used in this study. For each type, nine samples were measured.

**Table.** Compounds and Density of Examined Silicone Oils

Name	Compounds	Density at 25°C
Siluron 5000	100% Polydimethylsiloxane (5000 mPas)	0.97 g/cm^3^
Siluron 2000	95% Polydiemthylsiloxane (1000 mPas), 5% HMWC (2.5 mio mPas)	0.97 g/cm^3^
Siluron Xtra	90% Polydiemthylsiloxane (1000 mPas), 10% HMWC (2.5 mio mPas)	0.97 g/cm^3^
Densiron 68	30.5% F6H8, 69.5% Siluron 5000	1.06 g/cm^3^
Densiron Xtra	30.5% F6H8, 69.5% Siluron Xtra	1.06 g/cm^3^

### In Vitro Emulsions

Having applied a 2:8 ratio of silicone oil to BSS and preparing a 1-mL emulsion, one at a time, we followed the methodology previously described by Soós et al.^18^ for mixing Silicone oil and BSS as a hydrophilic phase using a Vortex stirrer for five minutes at 50% speed. We measured the physicochemical parameters of the emulsions immediately after the 5 minutes of mixing.

## Methods

### Shear Viscosity

The viscosimeter, ViscoQC 100 (Anton Paar GmbH, Graz, Austria) was used to measure the shear viscosity. Every sample was measured three times at 20°C, 25°C, 30°C, 35°C, and 37°C. To allow exact temperature measurement, a PTD-80 unit (Anton Paar GmbH) was connected to the viscosimeter. The stop-by time mode was used with a duration per measurement of 60 seconds with a shear rate of 100^−1^ s. A 60-second break was allowed in between measurements.

### Surface Tension

Surface tension was measured using an OCA 25 (DataPhysics Instruments GmbH, Filderstadt, Germany). The pendant drop method was used to determine the surface tension. The contours of drops of the sample are detected. An electronic dosage unit with three modules for syringes was used (DataPhysics Instruments GmbH). The oil was loaded into 1-mL syringes. The volume of one measurement corresponds with the maximum volume, which is held at the tip of the needle in the form of a drop. A needle with a diameter of 2.08 mm (12.5G) was used. We assumed a density of 0.97 g/cm^3^ at 25°C as specified by the oil manufacturer (Geuder AG). To account for possible interactions of the oil with the needle, for the first control oil, we checked if the calculated surface tension changes after 1, 2, and 3 minutes of holding a maximum droplet size at the tip of the needle. No meaningful changes occurred.

### Zeta Potential

The zeta potential of in vitro generated emulsions was measured using a Zetasizer Ultra (Malvern Instruments Ltd., Malvern, UK). The technique is based on electrophoretic light scattering. An electric double layer exists around the emulsion droplets. The more charges there are surrounding an emulsion droplet, the higher the electrical potential at this slipping plane. This electrical potential is referred to as the zeta potential. The higher the zeta potential, the more electrostatic repulsion is occurring between emulsion droplets of silicone oil in a hydrophilic phase. An emulsion with high absolute zeta potential is more stable than one with a lower value.

The zeta potential was measured at 37°C to simulate intraocular conditions. Every in vitro-generated emulsion was measured three times. The mean of the three measurements was used for analyses.

Before the initial start of the study, feasibility experiments were carried out with unused Siluron 5000 to test the emulsion stability after vortex mixing. Zeta-potential measurements were stable at less than 1, 3, and 6 minutes after inducing the emulsification by vortex mixing. A minimal time frame less than 30 seconds between vortex mixing and the start of the zeta potential measurement was chosen. Samples were prepared one by one to allow standardized timing of the zeta potential measurements. The zeta potential was immediately measured after the induction of in vitro emulsification of the explanted samples as well as the control samples. As such, changes in the zeta potential over time owing to a separation of the hydrophilic and lipophilic phases were ruled out as a potential confounder.

### Statistics

Statistical analyses were performed in STATA 16 and GraphPad Prism (GraphPad Software Inc., San Diego, CA). *P* values of less than 0.05 were considered statistically significant. Unpaired *t* tests and Mann–Whitney *U* tests were performed, as appropriate.

## Results

### Patient Characteristics

The 15 samples of explanted silicone oil had been implanted for between 30 and 506 days (mean, 196 days). All patients had undergone pars plana vitrectomy to treat retinal detachment which required the implantation of silicone oil as a long-term endotamponade. Five (33.3%) of the 15 patients were female and 3 (20%) of the 15 were diabetic. The mean patients age was 62 ± 9 years. One patient had severely elevated intraocular pressure after silicone oil tamponade.

### Shear Viscosity

**Figure 1.**
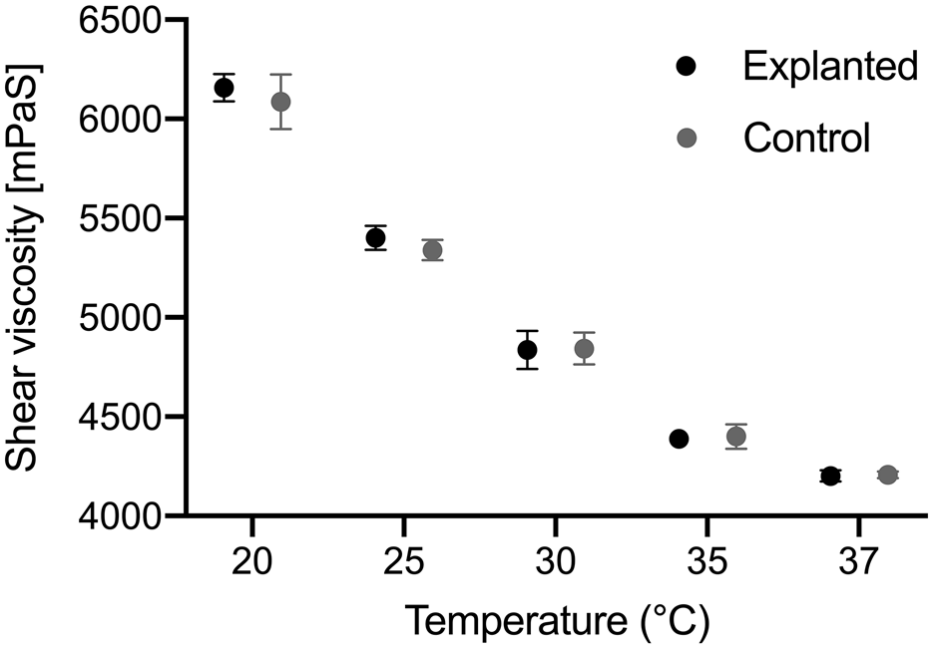
Mean viscosity ± SD of explanted silicone oil samples and the controls at 20°C, 25°C, 30°C, 35°C, and 37°C. A strong linear correlation between temperature and viscosity was found. There were no significant differences at any temperature level between explanted samples and the control group.

Figure 1 shows the mean viscosity of explanted and control samples at all temperature levels. At every temperature, no significant changes of shear viscosity between explanted samples and the control group were observed (multiple *t* tests, *P* = 0.68, *P* = 0.67, *P* = 0.98, *P* = 0.89, and *P* = 0.93 for 20°C, 25°C, 30°C, 35°C, and 37°C, respectively).

A linear relationship between temperature and viscosity was observed for all types of oil (r^2^ = 0.9871, r^2^ = 0.9975, r^2^ = 0.9952, r^2^ = 0.9693, and r^2^ = 0.9773 for Siluron 5000, Densiron Xtra, SiluronXtra, Siluron 2000, and Densiron 68, respectively; *P* < 0.01 for all).

### Surface Tension

**Figure 2.**
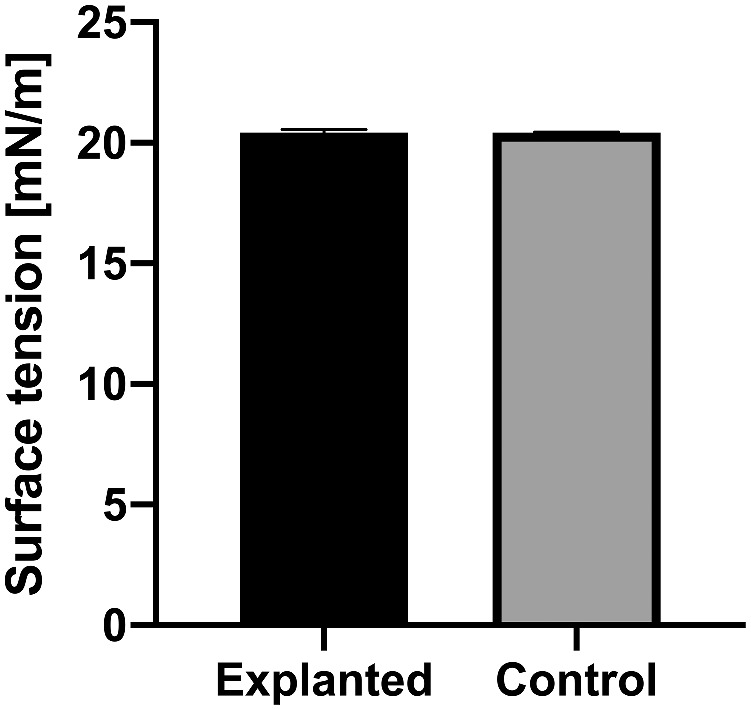
Mean surface tension ± SD of explanted silicone oil samples and unused new product, and the control group. There was no significant difference of surface tension at 25°C (*P* = 0.61) on the zeta potential and therefore emulsion stability. Graphs shows mean ± standard error of the mean.

The surface tension of explanted silicone oil samples did not significantly differ from the control group (*P* = 0.61, unpaired *t* test). Figure 2 presents the mean ± standard deviation values for both groups.

### Zeta Potential

**Figure 3.**
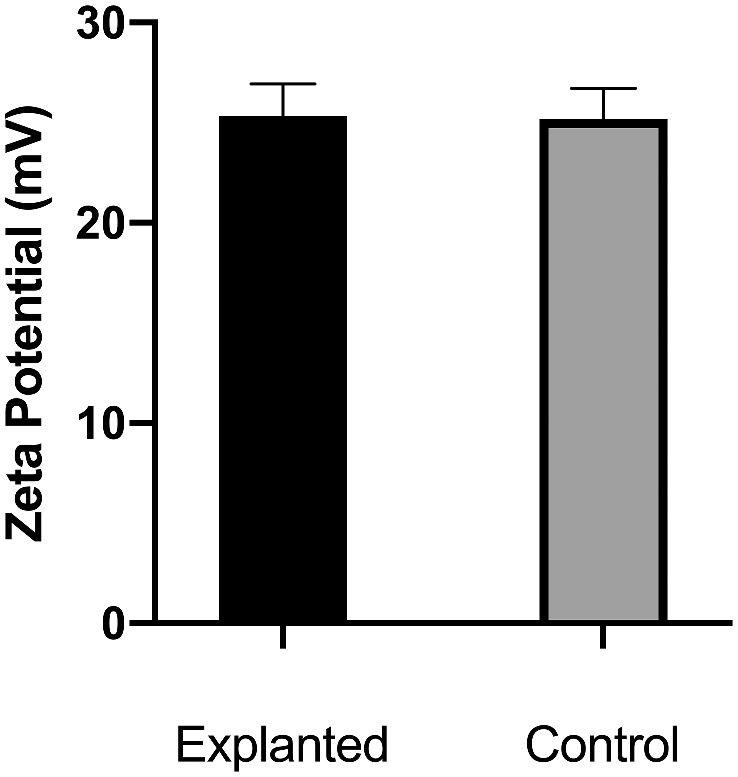
Zeta potential of in vitro generated emulsions of explanted samples and the control group. Mean and standard error of the mean of the zeta potential of in vitro generated zeta potential with BSS as the hydrophilic phase. No significant difference was found between explanted samples and controls (*P* = 0.84).

The zeta potential was measured to assess electrochemical stability of in vitro–generated emulsions. A higher absolute zeta potential within the same hydrophilic media, would speak for an increase an emulsion stability. No significant differences in the absolute value of zeta potential between explanted samples and control samples were found (*P* = 0.84, Mann–Whitney *U* test). Figure 3 showcases the absolute zeta potential for both.

**Figure 4.**
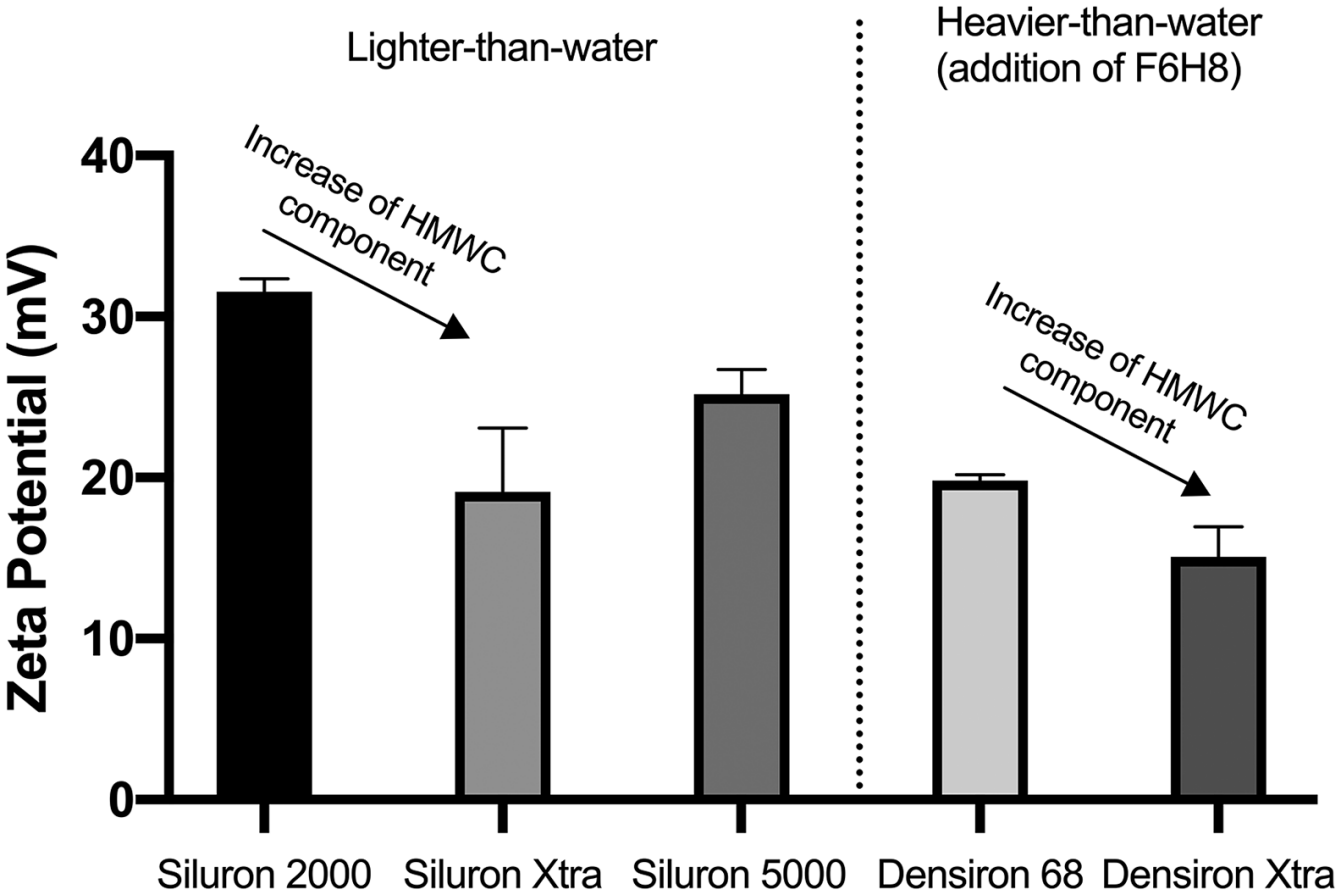
Zeta potential of in vitro generated emulsions of different oil types. Significant differences between the different oil types within the same hydrophilic media were measured suggesting the influence of chain length and oil composition on the zeta potential and therefore emulsion stability. Graphs shows mean ± standard error of the mean.

The zeta potential was also measured for Siluron 2000, Siluron Xtra, Densiron Xtra, and Densiron 68. The zeta potentials significantly varied between the different kinds of silicone oils (*P* < 0.01, *P* = 0.02, *P* < 0.0001, and *P* < 0.01 for Siluron 2000, Siluron Xtra, Densiron Xtra, and Densiron 68 compared with Siluron 5000, respectively). The mean and standard error of the mean are depicted in Figure 4.

## Discussion

We set out to assess whether silicone oil changes after its use as a long-term intraocular tamponade, and specifically we looked for changes in its physicochemical properties; shear viscosity, surface tension, and zeta potential. We compared the explanted oil samples with new, unused silicone oils and we focused on one kind of high-viscosity silicone oil, one that was common to 15 patients who presented with visible emulsified silicone oil: Siluron 5000, a 100% polydimethylsiloxane with a high purity. In addition to its availability in explanted samples, we had the rationale that this uniform formulation would allow a good comparison with a control (new and unused) group without bringing into question the possibility of changes of compound composition of the oil. Consequently, we recognize a limitation of our study. Further studies are needed to investigate whether there are changes of physicochemical parameters in other silicone oils, those of low viscosity and of high-viscosity oils with added HWMCs or added semifluorinated alkanes.

Many factors are suggested to influence the tendency of silicone oil to emulsify. One might be mechanical effects, such as the motion of the eye during saccades^12^^,^^19^ and another factor might be the recently described mechanism of the break-up of silicone oil^11^^,^^13^^,^^20^^,^^21^ that allows increased adherence to the ocular tissue, thus promoting the formation of emulsion droplets. Biological surfactants derived from ocular tissue and blood (eg, fibrinogen, fibrin, γ-globulin, very-low-density-lipoprotein, and alpha-1-glycoprotein),^22^ the amphiphilic properties of the retinal tissue itself,^23^ as well as the presence of lipophilic substances increasingly contaminating the silicone oil; these are all implicated in influencing the rate of emulsification.

There are relatively few in vivo data on the changes in silicone oil when exposed to biological environment in the eye for extended durations of time. Brunner et al.^14^ and Lakits et al.^4^ assessed the stability of silicone oils after prolonged clinical use and concluded that although the oils are chemically stable, they are not biologically inert. They observed changes of low-molecular-weight components in different high-viscosity silicone oils, whereas lipophilic molecules like cholesterol increased in concentration over time. Other studies show the increase of other lipophilic substances that are not present in the physiological vitreous cavity: Silicone oil is known to dissolve retinol,^17^ alpha-tocopherol,^16^ and fatty acids from ocular tissue.^15^ Certain surfactants are partly soluble in silicone oil, and in an in vitro study, Lu et al.^24^ showed the effects on the oil's surface tension of these surfactants.

However, none of these studies researched the physicochemical parameters of explanted silicone oil, which could be influenced owing to the dissolution of lipophilic substances, breakdown of silicone oil, or contamination of the oil with amphiphilic proteins.^18^^,^^24^

Amphiphilic substances within the eye and blood have both hydrophilic and hydrophobic parts that can change the properties of the interface between silicone oil and a hydrophilic phase. A decreased surface tension may promote oil emulsification, in this case, a decreased interfacial tension between hydrophilic intraocular media and silicone oil.^25^^,^^26^ In fact, the retina itself displays both hydrophilic and lipophilic properties as shown by Rubowitz et al. in 2020.^23^ Based on Antonoff's rule, which states that the surface tension at the interface between two saturated liquid layers in equilibrium is equal to the difference between the individual surface tensions of similar layers when exposed to air, and later modifications to this rule: lower surface tension values can translate to a lower interfacial tension between silicone oils and biological hydrophilic media in the eye with an increased risk of emulsification. In our study, we did not see significant changes of surface tension of silicone oil after long-term intraocular use. This phenomenon could be explained by the high shear viscosity of Siluron 5000. The oil's viscosity might influence the surface energy needed for the dispersion of one liquid into the other, affecting the speed and the extent of the emulsification process. In an in vitro study, Lu et al.^24^ demonstrated the influence of surfactants on emulsification in an eye-on-a-chip setup within 4 days of constant saccades using a low viscosity silicone oil (100). Lu's group showed that the interfacial tension between the silicone oil and hydrophilic media is influenced in a dose-dependent fashion based on the used surfactant, leading to more emulsion droplets over time. However, some of the examined surfactants did increase the number of emulsion droplets without changing interfacial tension.

Another explanation for why we did not see changes in surface tension might be the solubility of the biological surfactants in silicone oil. We focused on the silicone oil itself. In vitro, the solubility of surfactants in the silicone oil determined the number of silicone oil-in-water droplets that could be found, which is the most common form of emulsion we see in patients.^27^ Shinoda^28^ emphasized the hydrophilic–lipophilic balance value of biological amphiphilic molecules might be tending toward the hydrophilic phase rather than the silicone oil. In this study, only the lipophilic silicone oil phase after the removal was analyzed. In practice, this practice removes a variety of factors present at the important interface layer between the silicone oil and thin aqueous film between the oil and the retina. Thus, although in vitro after explantation we see no significant changes in parameters influencing the oil's tendency to emulsify, in vivo many molecules and factors can still strongly influence the oil–retina watery interface, weakening the oil's tamponing efficacy. Although it was the goal of our study to rule out potential changes to the lipophilic phase only, the in vivo environment as a whole, including the hydrophilic and lipophilic phase as well as its interface, has to be considered.

Although an impaired interfacial tension can be used as a proxy for the rate of occurrence of emulsion droplets, the zeta potential is one measure of emulsion stability.^29^ It is based on the electric double layer, made up of counter-ions of opposite charges that surround each emulsion droplet. These counter-ions may move with the droplet, developing a slipping plane beyond the emulsion droplet. The induced electrical potential is commonly referred to as the zeta potential.^18^ The absolute value of the zeta potential is a measure of the electrochemical stability of emulsion droplets. Amphiphilic substances within the eye could function as an electrostatic or steric barrier against the coalescence of droplets, thus increasing emulsion stability.

Again, as with our results on surface tension, we could not detect a significant difference in the zeta potential in explanted silicone oil samples compared with new, unused silicone oil. Again, one might explain this result if it is likely that most of the surfactants are in solution in the aqueous rather than the silicone oil to the extent that there is not a sufficient number of amphiphilic molecules in solution in the oil needed to show significant changes of emulsion stability. Further in vitro studies are needed to elucidate the effects on emulsion stability of biological emulsifiers found in the eye. We evaluated the electrochemical emulsion stability of oils with different viscosity, added HWMCs and added perflourhexyloctane (F_6_H_8_). The addition of HWMCs in different compositions (e.g., 5% or 10% of a 2.5 million mPas component in Siluron 2000 and Siluron Xtra, respectively) to a low-viscosity silicone oil (1000 mPas) decreased emulsion stability. To our knowledge, this study is the first of the electrochemical emulsion stability of these silicone oils. However, our data are in line with results from Lu et al.^11^ and Caramoy et al.^30^ The addition of HWMCs showed beneficial effects on the rate of emulsification and decreased adherence to ocular tissue, thus decreasing the number of emulsion droplets formed. The lower electrochemical stability could allow the eased confluence of the emulsion droplets, therefore lowering complications caused by emulsification. Further studies are needed to investigate the emulsion stability of silicone oils with high-molecular-weight compounds and possible interaction with hydrophilic media in the eye which could act as surfactants.

We also examined the combination of Siluron 5000 with F_6_H_8_ Densiron 68; and Siluron Xtra with F_6_H_8_: Densiron Xtra. Again, these compound oils with HMWC components showed a decreased electrochemical emulsion stability. This finding is in line with data presented in 2015 by Caramoy et al.: They showed that the increasing the percentage of the HMWC-component decreased the emulsification measured by the emulsified area after applying sonic waves. Densiron Xtra showed a smaller electrochemical emulsion stability when compared with Densiron 68. This finding is in line with the fact that Siluron Xtra with its 10% HMWC showed a smaller zeta potential when compared with Siluron 5000*.* Although the conductivity of the examined polydimethylsiloxane with varying chain length is comparable, this is not the case for silicone oils with added semifluorinated alkanes. As such, silicone oils with this add-on cannot be directly compared with oils purely consisting of polydimethylsiloxane.

Finally, we measured the viscosity of the explanted oils to establish whether it had increased during implantation. It is known that when a nearly stable water-in-oil emulsion is formed, the emulsified droplets may increase the viscosity of the oil phase.^18^ Again, we did not note any significant differences. This finding is in line with most of the clinical studies^5^^,^^31^ that describe oil-in-water emulsions more frequently than water-in-oil emulsions.

Our results are from only one brand of single-compound high-viscosity silicone oil. Low-viscosity silicone oil may behave differently. Also, the impact of different quality standards should be examined. Our explanted samples had remained within the eye for a mean of 196 days, which we consider is a clinically meaningful mean timeframe, but future studies should examine the physicochemical parameters of silicone oil removed from the eye after more extended periods. Also, we used vortex mixing as our primary method of inducing emulsification. This holds inherent limitations with droplet size distribution and a possibly higher polydispersity index. Further studies should consider other methods of inducing emulsification, such as homogenization and sonification*.*

## Conclusions

Our results indicate that the eye's posterior chamber provides the oil with constant proximity to hydrophilic tissue and media, but this biological environment does not influence the physicochemical parameters and emulsion stability of high-viscosity silicone oil. Effects mediated by the contamination of silicone oil with lipophilic and amphiphilic molecules may not be relevant during a typical duration of an ophthalmic endotamponade. Nevertheless, further studies are needed to explain the role of amphiphilic molecules dissolved in the hydrophilic media at the oil interface.
